# Macrocephaly and Characteristic MRI Findings as Early Clues to a Hereditary Overgrowth Syndrome

**DOI:** 10.7759/cureus.101863

**Published:** 2026-01-19

**Authors:** Catarina Cezanne, Kaylene Freitas, Susana L Ferreira, Ana M Queiroz, José P Monteiro

**Affiliations:** 1 Paediatrics, Unidade Local de Saúde Almada-Seixal, Almada, PRT; 2 Paediatrics, Hospital Dr. Nélio Mendonça, Sesaram-Eperam, Funchal, PRT; 3 Genetics, Instituto Português de Oncologia Francisco Gentil, Lisbon, PRT

**Keywords:** cancer surveillance, developmental delay in childhood, genetic diagnosis, germline pathogenic variants, macrocephaly, overgrowth syndrome, pediatric brain mri, perivascular spaces, pten hamartoma tumor syndrome (pths)

## Abstract

Macrocephaly may be the presenting feature of underlying genetic conditions in childhood. We report a five-year-old boy with persistent macrocephaly above +3 standard deviations since infancy and subtle facial dysmorphisms. Neurological examination revealed hypotonia, a wide-based gait, and fine and gross motor difficulties. Developmental assessment confirmed psychomotor delay without features of autism spectrum disorder. Brain MRI revealed multiple enlarged perivascular spaces involving the bilateral subcortical white matter and the corpus callosum, as well as callosal thickening. Genetic testing identified a heterozygous likely pathogenic variant in the PTEN gene, with maternal transmission confirmed on familial testing. Timely genetic diagnosis allows appropriate genetic counselling and clinical follow-up.

## Introduction

Macrocephaly is a common reason for referral to a paediatric clinic. In most cases, the underlying cause is benign and familial. However, not all inherited forms of macrocephaly are benign, and a subset of children requires further investigation. In particular, a markedly increased occipitofrontal circumference (OFC) more than +2 standard deviations (SD) above the age- and sex-matched mean (or above the 97th percentile), especially when non-familial, should prompt a structured diagnostic work-up that typically includes brain MRI [1,2]. In early childhood, an increased OFC may be the only obvious clinical finding, and in this context, it has become one of the key indicators to consider germline testing of the PTEN gene and, consequently, a diagnosis of PTEN hamartoma tumour syndrome (PHTS). Several paediatric series have reported macrocephaly in almost all children with confirmed PTEN pathogenic variants, and in many of them it represents the first or most prominent sign that brings the child to medical attention [2].

All individuals with a molecularly confirmed PTEN pathogenic variant carry an increased lifetime risk for both benign and malignant tumours and therefore should be enrolled in dedicated cancer surveillance programmes [2-4]. Importantly, thyroid carcinoma has been reported even in young children, underscoring the need for timely recognition of PHTS and early implementation of screening protocols [3-5]. Therefore, an early diagnosis is crucial to allow appropriate follow-up, targeted surveillance and preventive strategies for the child and, when relevant, affected family members. With this case, we aim to draw attention to PHTS as a potential diagnosis in children with macrocephaly and to the characteristic abnormalities that can be identified on brain MRI. Since MRI is often part of the standard work-up for macrocephaly, recognising these imaging features may provide a valuable radiological clue that leads to earlier genetic confirmation and initiation of tumour surveillance and family counselling.

## Case presentation

**Figure 1:**
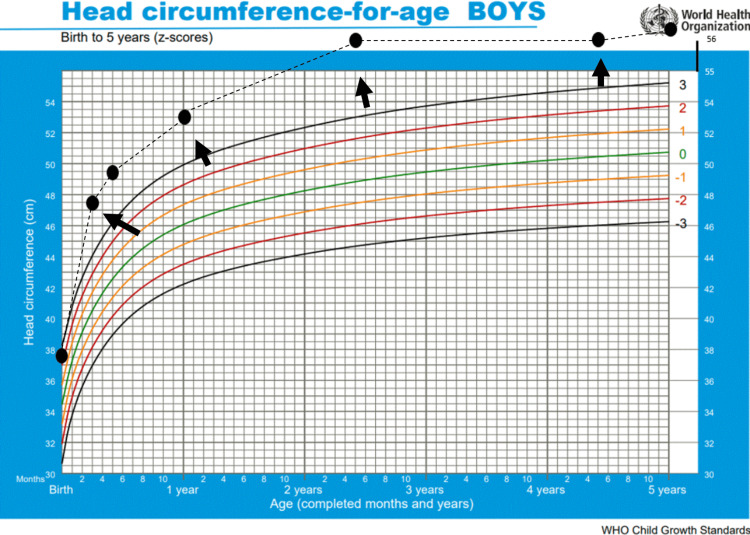
The WHO head circumference-for-age chart from birth to five years of age Our patient’s head circumference consistently tracked above +3 standard deviations from infancy onward, indicating persistent macrocephaly. This chart is sourced from the WHO database on 'head circumference for age' [6].

We report the case of a five-year-old boy who was followed at our outpatient clinic for macrocephaly above +3 SD, with onset in infancy (Figure 1). He was born following an uncomplicated pregnancy with regular antenatal care and a full-term eutocic delivery. At birth, he weighed 4515 g (>+2 SD), measured 54 cm (>+2 SD), and had a head circumference of 37.7 cm (>+2 SD). His family history was notable for a maternal head circumference of 62 cm (+6.2 SD) and macrosomia.

On physical examination, the patient exhibited facial dysmorphisms, including frontal bossing, mild hypertelorism, almond-shaped eyes, and a broad, depressed nasal bridge. Growth parameters over the first five years of life showed that weight stabilised between the 15th and 50th percentiles and height at the 15th percentile, while head circumference remained increased at 56.5 cm (>+3 SD). Papillomatous lesions were also observed on the left palm. Neurological examination revealed hypotonia, decreased deep tendon reflexes, a wide-based gait, and difficulties with coordination and fine motor skills. Formal developmental assessment using the Griffiths III scale (at 60 months) demonstrated psychomotor developmental delay, with greater impairment in coordination, fine and gross motor skills, and comprehension of complex verbal information. No features suggestive of autism spectrum disorder were identified.

**Figure 2:**
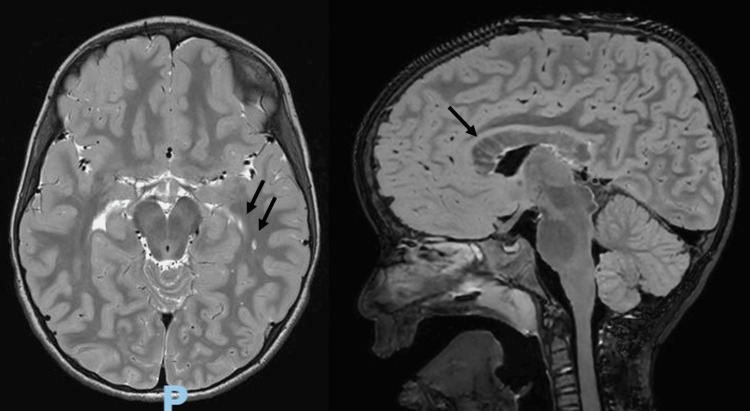
Enlarged perivascular spaces and callosal thickening on brain MRI Left panel: Brain MRI showing multiple enlarged perivascular spaces (Virchow-Robin spaces) involving the bilateral cerebral subcortical white matter and the entire length of the corpus callosum. Right panel: The corpus callosum is thickened, with the exception of the splenium, which is of normal thickness.

The brain MRI revealed multiple enlarged perivascular spaces (Virchow-Robin spaces) involving the bilateral subcortical white matter and the entire corpus callosum (Figure 2, left panel), as well as thickening of the corpus callosum (>97th percentile), except for the splenium, which was of normal thickness (Figure 2, right panel). These findings are consistent with the spectrum of imaging abnormalities described in PHTS. 

A macrocephaly gene panel based on next-generation sequencing was requested and revealed a heterozygous frameshift variant in PTEN (NM_000314.8): c.641dup (p.Phe215Valfs*28), classified as likely pathogenic according to the American College of Medical Genetics and Genomics and the Association for Molecular Pathology criteria. The patient’s mother carried the same variant.

## Discussion

The PTEN hamartoma tumour syndrome encompasses a group of clinical overgrowth disorders caused by autosomal-dominant germline pathogenic variants in the tumour suppressor gene PTEN [2,7]. These conditions have multiple overlapping features and include Cowden syndrome, Bannayan-Riley-Ruvalcaba syndrome, Lhermitte-Duclos disease, and Proteus-like phenotypes. In addition, juvenile polyposis of infancy, autism spectrum disorder associated with macrocephaly, and children presenting with macrocephaly and developmental delay and/or cognitive impairment have all been linked to PTEN pathogenic variants [2,5]. Despite including several related syndromes, PHTS is rare, with an estimated prevalence of 1:200,000-250,000 individuals [3]. In paediatrics, diagnosis is particularly challenging, as the existing diagnostic criteria, largely developed for Cowden syndrome, are often insufficient, contributing to under-recognition of the condition [2,8,9]. In early childhood, macrocephaly may represent the only or most prominent clinical manifestation.

In cases of macrocephaly associated with additional clinical findings such as suggestive dysmorphic features or autism spectrum disorder, genetic evaluation of the PTEN gene is recommended [1,10]. In 2011, a paediatric clinical scoring system was proposed to guide PTEN testing, requiring macrocephaly (≥ +2 SD) together with at least one of the following: autism spectrum disorder or developmental delay; typical dermatological features such as lipomas, trichilemmomas, oral papillomas, or penile freckling; vascular anomalies such as arteriovenous malformations or haemangiomas; or gastrointestinal polyps [8].

Because macrocephaly may be the only or most evident clinical sign in young children, brain MRI is often included in the diagnostic evaluation to investigate potential structural abnormalities. Imaging findings may provide important diagnostic clues and help accelerate recognition of PHTS. In the present case, MRI demonstrated multiple enlarged perivascular spaces involving the bilateral subcortical white matter and the entire corpus callosum, as well as thickening of the corpus callosum. These findings have been previously described in association with PHTS [2,7,9,11]. Enlarged perivascular spaces and white matter abnormalities are frequently reported imaging features linked to PTEN mutations. Although this imaging pattern is not specific and may also be seen in mucopolysaccharidoses, oculocerebrorenal syndrome of Lowe, and hypomelanosis of Ito, the presence of which in a child with macrocephaly and developmental delay or autism spectrum disorder should strongly prompt PTEN testing [2,7,9].

As many pathognomonic clinical criteria of Cowden syndrome arise later in life, often during adulthood, MRI may contribute to earlier identification of affected children. Even when the full diagnostic criteria for Cowden syndrome are not met, individuals with germline PTEN pathogenic variants are believed to carry similar cancer risks. The most serious complications of PHTS relate to the markedly increased risk of breast, thyroid, endometrial, renal, and, less commonly, colorectal cancers. For this reason, cancer surveillance represents the most important aspect of management for any individual with a PTEN pathogenic variant [3,4,5,12].

Thyroid cancer is of particular relevance in paediatric patients, with cases reported as early as seven years of age. Consequently, thyroid ultrasound is recommended at the time of diagnosis, followed by annual imaging thereafter. Surveillance for other cancers begins in adulthood or five years before the youngest age of diagnosis of a PHTS-related malignancy in the family, whichever occurs first [3,4,5].

Genetic counselling for at-risk family members is essential, as PHTS is often underdiagnosed and follows an autosomal dominant inheritance pattern [3]. In the present case, maternal testing confirmed the familial variant, leading to the mother’s diagnosis and initiation of appropriate surveillance.

## Conclusions

This case illustrates how the combination of childhood macrocephaly, developmental findings, and characteristic MRI abnormalities can serve as a starting point for diagnostic evaluation of PHTS. Recognising these features is essential in paediatric practice, where typical syndromic manifestations may still be absent and diagnostic criteria are often insufficient. Early identification of PHTS enables timely initiation of surveillance, appropriate genetic counselling, and assessment of at-risk relatives. Increasing awareness of these imaging and clinical clues among paediatricians, neurologists, and radiologists may help reduce diagnostic delays and ultimately improve long-term outcomes.
